# Using the *Xenopus* Developmental Eye Regrowth System to Distinguish the Role of Developmental Versus Regenerative Mechanisms

**DOI:** 10.3389/fphys.2019.00502

**Published:** 2019-05-08

**Authors:** Cindy X. Kha, Dylan J. Guerin, Kelly Ai-Sun Tseng

**Affiliations:** School of Life Sciences and Nevada Institute of Personalized Medicine, University of Nevada, Las Vegas, Las Vegas, NV, United States

**Keywords:** eye, apoptosis, retina, *Xenopus*, development, stem cells, regrowth, neural regeneration and repair

## Abstract

A longstanding challenge in regeneration biology is to understand the role of developmental mechanisms in restoring lost or damaged tissues and organs. As these body structures were built during embryogenesis, it is not surprising that a number of developmental mechanisms are also active during regeneration. However, it remains unclear whether developmental mechanisms act similarly or differently during regeneration as compared to development. Since regeneration is studied in the context of mature, differentiated tissues, it is difficult to evaluate comparative studies with developmental processes due to the latter’s highly proliferative environment. We have taken a more direct approach to study regeneration in a developmental context (regrowth). *Xenopus laevis*, the African clawed frog, is a well-established model for both embryology and regeneration studies, especially for the eye. *Xenopus* eye development is well-defined. *Xenopus* is also an established model for retinal and lens regeneration studies. Previously, we demonstrated that *Xenopus* tailbud embryo can successfully regrow a functional eye that is morphologically indistinguishable from an age-matched control eye. In this study, we assessed the temporal regulation of retinal differentiation and patterning restoration during eye regrowth. Our findings showed that during regrowth, cellular patterning and retinal layer formation was delayed by approximately 1 day but was restored by 3 days when compared to eye development. An assessment of the differentiation of ganglion cells, photoreceptor cells, and Müller glia indicated that the retinal birth order generated during regrowth was consistent with that observed for eye development. Thus, retina differentiation and patterning during regrowth is similar to endogenous eye development. We used this eye regrowth model to assess the role of known mechanisms in development versus regrowth. Loss-of-function studies showed that Pax6 was required for both eye development and regrowth whereas apoptosis was only required for regrowth. Together, these results revealed that the mechanisms required for both development and regrowth can be distinguished from regrowth-specific ones. Our study highlights this developmental model of eye regrowth as a robust platform to systematically and efficiently define the molecular mechanisms that are required for regeneration versus development.

## Introduction

Many animals have the ability to undergo regeneration, the successful restoration of tissues and organs after injury, but some animals lack this ability. Even though there is now considerable knowledge regarding the cellular and molecular pathways that regulate regeneration, the basic question of why the same tissues and organs from diverse (or even closely related) species often respond differently to injury and damage remains largely unanswered. To address this question, an area of focus has been to understand the role of developmental mechanisms in regeneration.

As regeneration requires the restoration of lost body structures generated during development, it is not surprising that a number of pathways involved in development are also active during regeneration (Schaefer et al., 1999; Lin and Slack, 2008; Malloch et al., 2009; Martinez-De Luna et al., 2011; Halasi et al., 2012; Meyers et al., 2012). However, it has been a challenge to effectively identify which developmental mechanisms are required for regeneration and to assess whether the roles of these mechanisms are similar or different during embryogenesis versus regeneration.

A second challenge in understanding the role of developmental mechanisms in regeneration is that existing models largely seek to examine regeneration in adult or mature differentiated tissues. The mature tissues are in contrast to a developmental environment where proliferation is high and cellular differentiation is low or just beginning. Furthermore, recent studies indicate that stem cells may have different functions in developing versus adult tissues (Wang and Conboy, 2010). Thus, it remains difficult to pinpoint the developmental mechanisms that can be successfully manipulated for inducing adult regeneration.

To address these challenges, a model to study regenerative mechanisms in the context of development is needed. This approach can reduce some of the complexities in comparing developmental processes to regenerative processes in mature tissues. For such a model to be valuable, two important characteristics would be needed: a high regenerative ability coupled with well-understood developmental events. *Xenopus laevis*, the South African clawed frog, fulfills these criterion as it is an animal that is an established and well-studied regenerative and developmental model (Beck et al., 2009; Sater and Moody, 2017). In particular, *Xenopus* eye development has been studied extensively (Perron and Harris, 1999; Rapaport, 2006; Henry et al., 2008; Viczian and Zuber, 2015). *Xenopus* can also regenerate mature eye tissues including the retina and lens [reviewed in Araki (2007), Vergara and Del Rio-Tsonis (2009), Henry et al. (2013), Tseng (2017)]. Additional advantages of the *Xenopus* system include: external development of embryos– facilitating developmental eye studies, amenability to molecular and cellular manipulations, and strong genetic similarity to humans. Using *Xenopus*, we established an embryonic model to study developmental eye regrowth (defined here as the ability of an embryo to compensate for missing tissues by restoring normal organ structures and function) (Kha and Tseng, 2018; Kha et al., 2018).

Our recent study showed that the *Xenopus* tailbud embryo at developmental stage (st.) 27 successfully regrew its eye after significant tissue loss (Kha et al., 2018). The completion of eye regrowth occurred by 4–5 days as overall development progressed without delay. Importantly, the regrown eye was age and size-appropriate with the expected complement of structures including the lens, retina, and pigmented epithelium. It was connected to the brain via the optic nerve and functional, displaying visual preference. Furthermore, the function of the regrown eye was dependent upon successful growth of new tissues since remnant eye cells in the regrowth-inhibited eyes lacked the ability to restore visual function (Kha and Tseng, 2018). To facilitate the use of this model to understand the role of developmental mechanisms in regrowth, we sought to determine whether eye formation during regrowth is comparable to endogenous eye development. Here, we show that while induction of regrowth delayed retinal differentiation and patterning, the overall retinogenesis process was consistent with a recapitulation of normal eye development. Furthermore, loss-of-function studies using our model showed that Pax6, a gene that is required for eye development, is also required for regrowth. In contrast, apoptosis is not required for eye development but is required for regrowth.

## Results

### Restoration of Cellular Patterning During Regrowth

In our previous study, histological analyses showed that retinal layer formation in a regrowing eye was delayed during the first 2 days post surgery (dps) even though overall development proceeded normally (Kha et al., 2018). The cellular patterning of the regrowing eye during this period was more similar to embryos at younger developmental stages. Notably, the regrowing eye regained overall size and cellular patterning comparable to an uninjured age-matched eye within 3–5 days post surgery (Kha et al., 2018). To better understand eye regrowth and assess this process as compared to normal eye development, we examined the temporal regulation of eye formation during regrowth at three successive 24-h timepoints.

**FIGURE 1:**
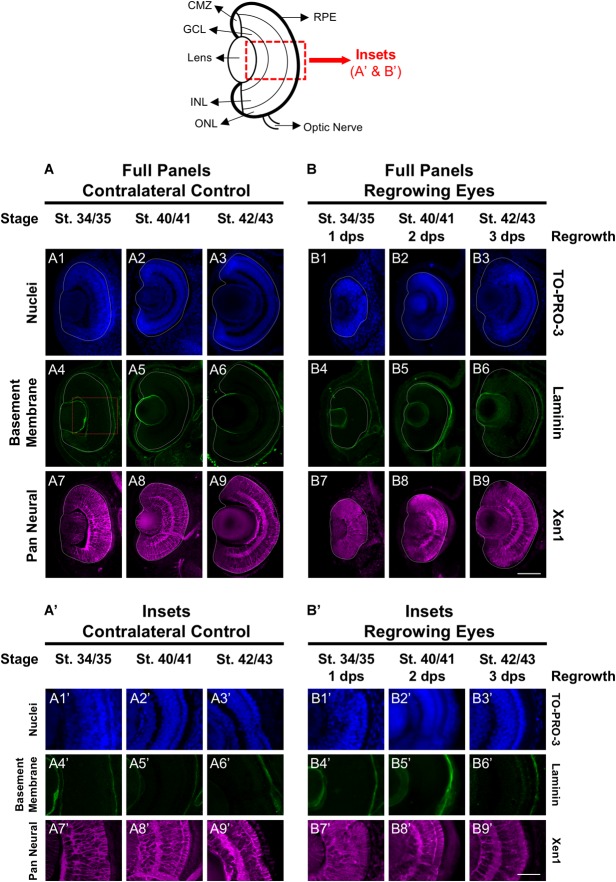
Regrown eyes regain cellular patterning by 3 dps. Images shown are immunostained, transverse sections at three developmental timepoints corresponding to 1, 2, and 3 days post surgery (dps). The top schematic is a diagram of a section through a mature, differentiated tadpole eye. **(A,B)** Regrowing eyes display retinal patterning comparable to the contralateral control eyes (unoperated) by 3 days. White dashed lines delineate each eye. **(A’,B’)** Representative images shown in panels **A’** and **B’** correspond to the region shown in the inset box in panel A4 for the corresponding A or B panel at high magnification. Blue color indicates nuclear staining (TO-PRO-3). Green color indicates the basal lamina (anti-Laminin), which is expressed in all basement membranes and outlines the optic vesicle. Magenta color indicates neural tissues (Xen1). Sample sizes: 1 day, *n* = 6; 2 days, *n* = 5; and 3 days, *n* = 5. **(A,B, A’,B’)** Up = dorsal, down = ventral, lens is on the left. Scale bar: **A,B** = 100 μm and **A’,B’** = 50 μm.

First, we assessed the overall cellular structure and patterning of the regrowing eye as compared to its uninjured contralateral eye. Here, we used the contralateral eye as the control to ensure that the comparative studies were made at the same developmental stages. Our previous work confirmed that the uninjured contralateral control was equivalent to the eye of age-matched sibling embryos [(Kha et al., 2018) and data not shown]. The lens and retina of the developing eye are surrounded by the basement membrane found in the extracellular matrix. To examine the basement membrane structure of the embryonic eye, a marker recognizing the basement membrane (an anti-Laminin antibody) was used (Kha et al., 2018). At st. 34/35 in the control embryonic eye, the basement membrane outlined the eye cup and the lens vesicle as it proceeds through development (Figure 1A4–6,A4’–6’). Induction of eye regrowth required tissue removal surgery, which also disrupted the basement membrane and showed lack of laminin expression (Kha et al., 2018). At 1 dps (st. 34/35), the basement membrane structure was restored as it surrounded the regrowing eye entirely. Similar to the control eye, the basement membrane surrounding the regrowing eye was maintained through to st. 42/43 as normal size is restored (Figure 1B4–6,B4–6’).

The Xen1 antibody recognizes neural tissues in the *Xenopus* embryo and is a reliable marker for visualizing retinal layers in the developing eye (Ruiz i Altaba, 1992; Kha et al., 2018). During *Xenopus* eye development, retinal layer formation begins at st. 33/34 and is completed by st. 41 (Holt et al., 1988). Consistent with previous studies, Xen1 expression showed that at st. 34/35, retinal layering was visible in the developing eye but not fully organized. Proper patterned retinal layers are seen by st. 40/41 (Figure 1A8, A8’). In contrast, a delay is observed during regrowth as Xen1 expression in the regrowing eye at st. 34/35 (1 dps) showed a lack of organization (Figure 1B7,B7’). By st. 40/41, the patterning in the regrowing eye is more similar to that of a younger control eye at st. 34/35 (compare Figure 1B8,B8’ with Figure 1A7, A7’). The retinal layer patterning in the regrowing eye was restored by 3 dps (st. 42/43) (Figure 1B9,B9’). Together, our data indicated that the basement membrane of the regrowing eye was fully restored by 1 dps, whereas retinal layer formation was delayed and then restored by 3 dps.

### Restoration of Retinal Differentiation During Regrowth

**FIGURE 2:**
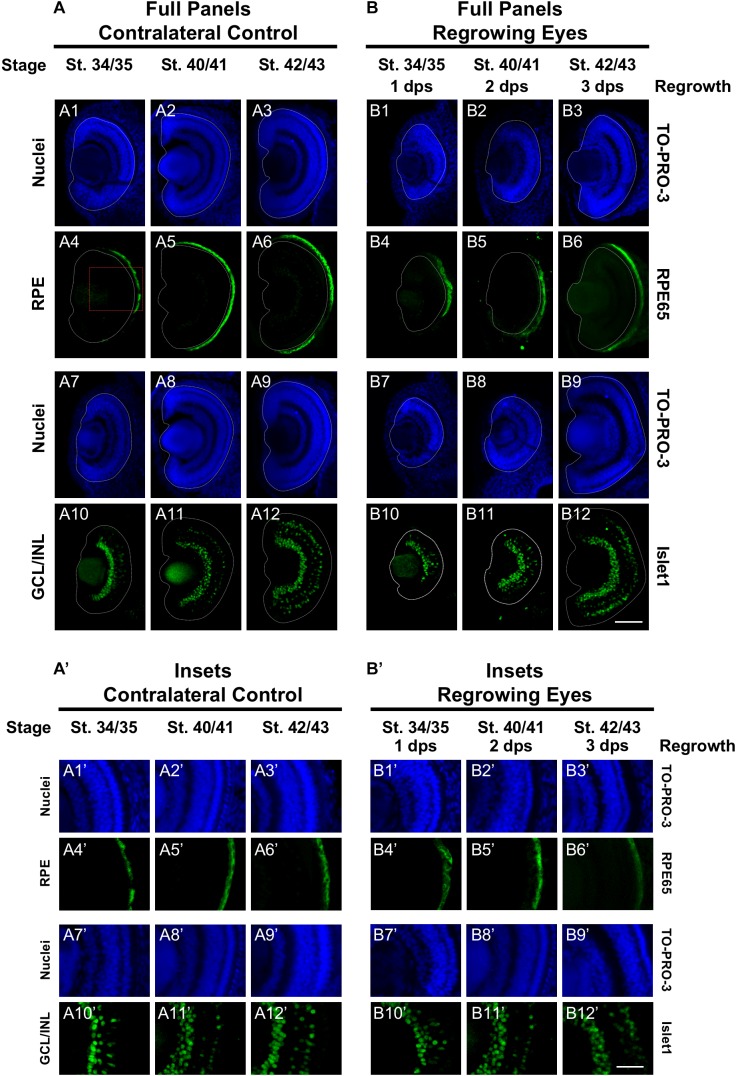
Regrown eyes regain retinal differentiation by 3 dps. Images shown are immunostained, transverse sections at three developmental timepoints corresponding to 1, 2, and 3 days post surgery (dps). **(A,B)** The contralateral control eyes (unoperated) complete retinogenesis by st. 41. By 1 dps, RPE is already visible in the regrowing eye as shown by anti-RPE65 signal (retinal pigmented epithelium; green). By 3 dps, Islet1 expression (identifying subpopulations of retinal ganglion cells and subsets of amacrine cells, bipolar cells, and horizontal cells; green) show expected retinal patterning of a mature eye. White dashed lines delineate each eye. **(A’,B’)** Images shown in panels **A’** and **B’** correspond to the region shown in the inset box in panel **A4** for the corresponding **(A or B)** panel at high magnification. Blue color indicates nuclear staining (TO-PRO-3). Sample sizes: 1 day, *n* = 5; 2 days, *n* = 7; and 3 days, *n* = 6. **(A,B, A’,B’)** Up = dorsal, down = ventral, lens is on the left. Scale bar: **(A,B)** = 100 μm and **(A’,B’)** = 50 μm.

The mature vertebrate retina is composed of the retinal pigmented epithelium (RPE) and the neural retina. For *Xenopus* eye development, retinal differentiation (retinogenesis) begins at st. 24 at the ventral midline and increasingly spreads toward the periphery along the presumptive retina (Holt et al., 1988). The process is completed by st. 41, when the differentiated structures found in a mature eye are present (Holt et al., 1988). This is a short window representing an overall period of approximately 2 days. The Xen1 expression patterns during regrowth indicated an initial delay in differentiation (Figure 1). We thus assessed the formation of the RPE and neural retina during regrowth. To assess RPE differentiation, we used an antibody against RPE65, a protein that is expressed in the mature RPE (Yoshii et al., 2007; Vergara and Del Rio-Tsonis, 2009). During eye development at st. 34/35, RPE65 was first expressed in a short segment extending from the ventral midline (Figure 2A4,A4’, white dashed lines demarcate the neural retina and lens). It was previously shown that retinal differentiation demonstrated a dorsal bias in maturity – dorsal cells in the central region differentiate slightly earlier than ventral ones (Holt et al., 1988). Indeed, RPE65 expression also showed a dorsal bias (Figure 2A4). By st. 40/41, RPE65 expression reached both the dorsal and ventral peripheries and remained the same at st. 42/43 (Figure 2A5–6,A5’–6’). During regrowth, RPE65 showed a similar expression pattern at 1 dps as the control (albeit larger) eye at the same stage (compare Figure 2B4,B4’ with Figure 2A4,A4’). This observation is consistent with our earlier finding that the black pigment of the RPE is morphologically visible by 1 dps in a regrowing eye (Kha et al., 2018). Unlike a control eye, RPE65 expression in the 2 dps regrowing eye failed to reach the periphery by st. 40/41 (compare Figure 2A5 with Figure 2B5). An additional day is required for the RPE65 expression to reach the periphery (Figure 2B6). Together, the data indicate that RPE differentiation was delayed as compared to the control eye. However, RPE differentiation was restored by 3 days as the embryo reached the mature eye stage (st. 42/43).

Next, we examined retinal differentiation during regrowth. The neural retina consists of three nuclear layers and two plexiform layers (Figure 1: schematic shows the 3 nuclear layers). Photoreceptor cells (rods and cones) are located in the outer nuclear layer (ONL). Bipolar, horizontal, and amacrine cells are found in the inner nuclear layer (INL). The retinal ganglion cells are located in the ganglion cell layer (GCL). The birth order of retinal cell types occur in a consistent yet overlapping temporal order with the retinal ganglion cells (RGCs) being the first to be specified, followed by horizontal cells, cone photoreceptor cells, rod photoreceptor cells, amacrine cells, bipolar cells, and lastly the Müller glial cells (Wong and Rapaport, 2009). Using known antibody markers that identify retinal cell types, we assessed the timing of retinogenesis.

Islet1 is a marker of vertebrate RGCs including *Xenopus* (Dorsky et al., 1997). The Islet1 antibody that we used also identified additional cells in the INL including subsets of amacrine, bipolar, and horizontal cells (Álvarez-Hernán et al., 2013). At st. 34/35, the presumptive GCL was readily apparent and somewhat patterned in the control eye (Figure 2A10,A10’). At this stage, a small number of differentiated cells in the presumptive INL showed Islet1 expression. The number of Islet1-positive cells in the INL increased with increasing age (Figure 2A10–12,A10’–12’). At 1 dps (st. 34/35) in the regrowing eye, the presumptive RGC layer is apparent but was poorly patterned and remained incomplete at the periphery as compared to the control eye (compare Figure 2B10,B10’ with Figure 2A10,A10’). At 2 dps (st. 40/41), the RGC layer has reached the periphery with some Islet1-positive cells found in the INL but remained less patterned than the same stage control (compare Figure 2B11,B11’ with Figure 2A11,A11’). At 3 dps, the Islet1 expression pattern was largely comparable to the control eye (compare Figure 2B12,B12’ with Figure 2A12,A12’). Together, the data showed that retinal differentiation was delayed as compared to the control eye. However, retinal differentiation was restored by 3 days as the embryo reached the mature eye stage (st. 42/43).

### Restoration of Cone Photoreceptor Differentiation

**FIGURE 3:**
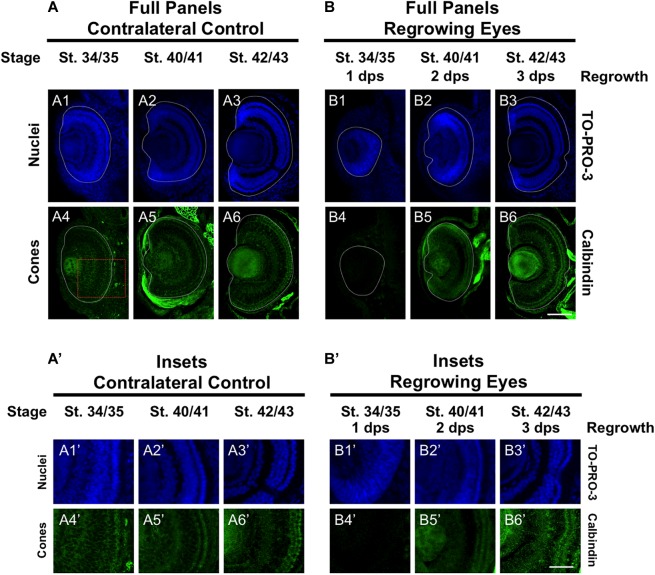
Regrown eyes regain cone differentiation by 3 dps. Images shown are immunostained, transverse sections at three developmental timepoints corresponding to 1, 2, and 3 days post surgery (dps). **(A,B)** Differentiation of cone photoreceptor cells is delayed during 1, 2 dps but regains patterning that is comparable to contralateral control eyes (unoperated) by 3 days. White dashed lines delineate each eye. **(A’,B’)** Images shown in panels **A’** and **B’** correspond to the region shown in the inset box in panel **A4** for the corresponding **A or B** panel at high magnification. Blue color indicates nuclear staining (TO-PRO-3). Green color indicates anti-Calbindin signal (cone photoreceptors). Sample sizes: 1 day, *n* = 6; 2 days, *n* = 5; and 3 days, *n* = 6. **(A,B, A’,B’)** Up = dorsal, down = ventral, lens is on the left. Scale bar: **A,B** = 100 μm and **A’,B’** = 50 μm.

To further define the temporal delay in retinal differentiation during regrowth, we used an anti-Calbindin antibody to assess cone photoreceptor differentiation as we had done previously (Kha et al., 2018). In *Xenopus*, both cone and rod photoreceptors are generated at similar times in the middle of the retinal differentiation sequence. However, a close study of retinogenesis indicated that cone photoreceptors are generated just prior to rod photoreceptors and are the 3rd cell type to be specified (Wong and Rapaport, 2009). During eye development at st. 34/35, a few cone photoreceptor cells were detected by calbindin expression in the central region of the presumptive photoreceptor layer (Figure 3A4,A4’). By st. 40/41, cone photoreceptor differentiation reached the retinal periphery and appeared to be restored (Figure 3A5,A5’). This pattern was maintained in st. 42/43 (Figure 3A6,A6’). In contrast, cone photoreceptors were not observed in the regrowing eye at 1 dps (st. 34/35; Figure 3B4,B4’). As regrowth proceeded, cone photoreceptor differentiation was visible by 2 dps and showed patterning that is somewhat comparable to age-matched developing eye (compare Figure 3B5,B5’ with Figure 3A5,A5’). By 3 dps, cone photoreceptor cells have expanded along the retina and showed a comparable pattern to the control eye at st. 42/43 (compare Figure 3B6,B6’ to Figure 3A6,A6’). Our results indicated that in the regrowing eye, cone photoreceptor cell differentiation is delayed by 1 day but is restored by 3 days when the embryo reached the mature eye stage (st. 42/43).

### Restoration of Rod Photoreceptor Differentiation

**FIGURE 4:**
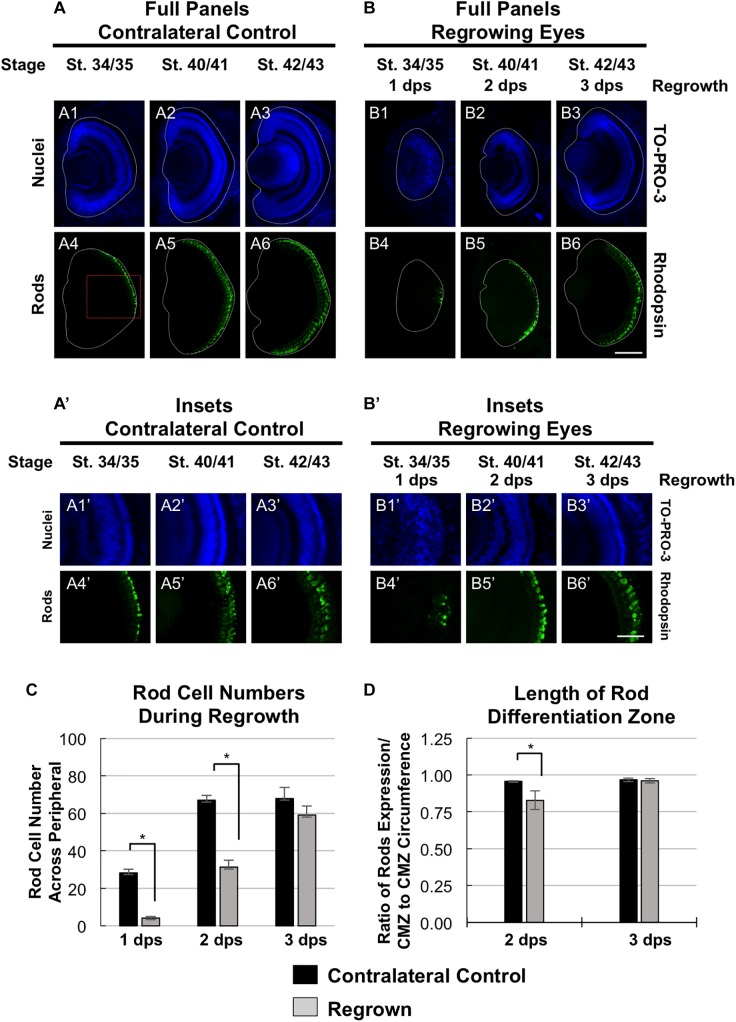
Regrown eyes regain rod differentiation by 3 dps. Images shown are immunostained, transverse sections at three developmental timepoints corresponding to 1, 2, and 3 days post surgery (dps). **(A,B)** Differentiation of rod photoreceptor cells is delayed during 1, 2 dps but regains patterning that is comparable to contralateral control eyes (unoperated) by 3 days. White dashed lines delineate each eye. **(A’,B’)** Images shown in panels **A’** and **B’** correspond to the region shown in the inset box in panel **A4** for the corresponding **A or B** panel at high magnification. Blue color indicates nuclear staining (TO-PRO-3). Green color indicates anti-Rhodopsin signal (rod photoreceptors). Sample sizes: 1 day, *n* = 5; 2 days, *n* = 7; and 3 days, *n* = 7. **(A,B, A’,B’)** Up = dorsal, down = ventral, lens is on the left. Scale bar: **A,B** = 100 μm and **A’,B’** = 50 μm. **(C)** Quantification of rod photoreceptor cells in the regrowing eye structure at three developmental timepoints corresponding to 1, 2, and 3 dps. The number of rod photoreceptors per 60 μm section in the regrown eye is comparable to number of rod photoreceptor cells in the contralateral control eyes by 3 dps. ^∗^denotes *p* < 0.05 (*n* > 5 per timepoint). Data are means ± SEM. **(D)** Rod photoreceptor cells expression pattern was measured and compared to the overall circumference of the retinal layer from one end of the ciliary margin zone (CMZ) to the end of the opposite CMZ in both regrowing and contralateral eyes. The ratio of rhodopsin expression in the retinal layer over the retinal layer circumference measurements is shown. By 3 dps, the rod photoreceptor cell expression is comparable to the contralateral control eye. ^∗^denotes *p* < 0.05 (*n* > 6 per timepoint). Data are means ± SEM.

To further define the temporal delay in retinal differentiation during regrowth, we assessed rod photoreceptor differentiation using anti-Rhodopsin antibody (Kha et al., 2018). Rod photoreceptor cells are the fourth of seven retinal cell types to be specified (Wong and Rapaport, 2009). At st. 34/35, rod photoreceptor cells were first seen in a short segment extending from the ventral midline (Figure 4A4,A4’), reached the periphery by st. 40/41 and maintained at st. 42/43 (Figure 4A5,6,A5’,6’). In contrast, there were very few rod photoreceptor cells seen in the ventral midline in the regrowing eye at 1 dps (st. 34/35; Figure 4B4,B4’). This was in contrast to the formation of GCL, which appeared to be more advanced at the same stage (compare Figure 4B4,B4’ with Figure 2B10,B10’). As regrowth proceeded, rod photoreceptor differentiation expanded along the retina until it showed a similar pattern to the control eye by st. 42/43 (compare Figure 4B6,B6’ with Figure 4A6,A6’).

To confirm our observations, we quantitated and compared the number of rod photoreceptor cells during development and regrowth (Figure 4C). At 1 dps, there were 28.3 ± 1.8 rod photoreceptor cells in the control eye whereas there were only 4.2 ± 0.8 rod photoreceptor cells in the regrowing eye (*n* > 5 per condition and timepoint, *p* < 0.05). At 2 dps, the number of rod photoreceptor cells in the control eye increased to 67.0 ± 2.7 whereas the number of rod photoreceptor cells in the regrowing eye only reached 31.3 ± 3.8. By 3 dps, there were 69.6 ± 5.4 rod photoreceptor cells in the control eye whereas there was a significant increase in the regrowing eye to 55.5 ± 5.4 rod photoreceptor cells. Measurements of the length of the rod differentiation zone supported the rod photoreceptor cell counts (Figure 4D). At 2 dps, the rod differentiation zone was shorter in the regrowing eye as compared to the control eye (*n* > 5 per condition, *p* < 0.05). However, by 3 dps, the rod differentiation zone in the regrowing eye reached comparable length to the uninjured control eye (*n* > 6 per condition, *p* = 0.73). Together, the data showed that rod photoreceptor differentiation and patterning was delayed as compared to the control eye. However, rod photoreceptor differentiation was restored by 3 days as the embryo reached the mature eye stage (st. 42/43). Combined, the progress of RGC differentiation at 1 dps as compared to the initial lack of rod photoreceptor differentiation at the same timepoint also suggested that the developmental retinal birth order is maintained during regrowth.

### Restoration of Müller Glial Cell Differentiation

**FIGURE 5:**
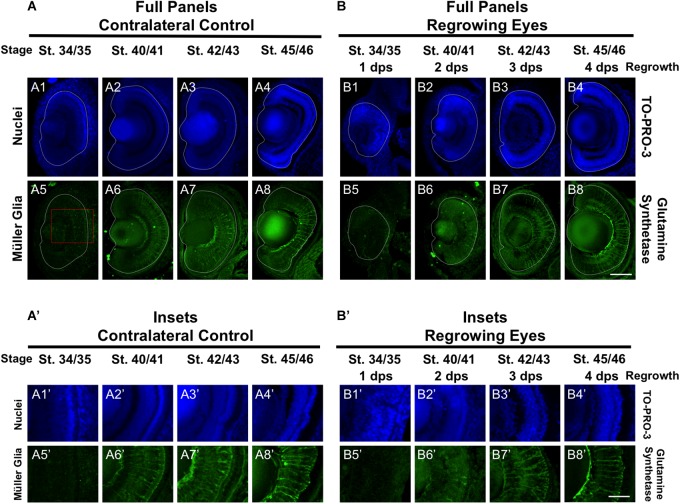
Regrown eyes regain Müller glia differentiation by 4 dps. Images shown are immunostained, transverse sections at four developmental timepoints corresponding to 1, 2, 3, and 4 days post surgery (dps). **(A,B)** Regrown eyes show differentiation of Müller glial cells beginning at 2 dps. However, proper patterning of Müller glial cells is delayed in the regrowing eyes when compared to the contralateral control eyes until 4 dps. White dashed lines delineate each eye. **(A’,B’)** Images shown in panels **A’** and **B’** correspond to the region shown in the inset box in panel **A5** for the corresponding **A or B** panel at high magnification. Blue color indicates nuclear staining (TO-PRO-3). Green color indicates anti-Glutamine Synthetase (identifies Müller glial). Sample sizes: 1 day, *n* = 5; 2 days, *n* = 5; and 3 days, *n* = 5. **(A,B, A’,B’)** Up = dorsal, down = ventral, lens is on the left. Scale bar: **A,B** = 100 μm and **A’-B’** = 50 μm.

In the retina, the Müller glial cells serve as neuronal support cells. They are typically the last retinal cell type to be specified (Holt et al., 1988). Our data on RGC and rod photoreceptor differentiation during regrowth were consistent with the maintenance of the developmental retinal birth order (Figure 2, 4). We hypothesized that if retinal differentiation during regrowth is similar to developmental retinal differentiation, then the cellular patterning of Müller glial cells would be the last to be restored. To test our hypothesis, we used a Müller glial cell marker, an anti-glutamine synthetase antibody, to assess its differentiation pattern as we did previously (Kha et al., 2018). As expected for a cell type that is the last to be specified during retinogenesis, there was no detectable glutamine synthetase expression indicative of Müller glial differentiation in the control eye at st. 34/35 (Figure 5A5,A5’). The presence of Müller glial cell patterning was visible by st. 40/41 and full patterning was observed by st. 42/43 (Figure 5A6,7,A6’,7’). In the regrowing eye, there was also no detectable Müller glial differentiation at st. 34/35 (Figure 5B5,B5’). By 2 dps (st. 40/41), only a small number of Müller glial cells were visible – much less when compared to the control eye (compare Figure 5B6,B6’ with Figure 5A6,A6’). By 3 dps (st. 42/43), the pattern in the regrowing eye was similar to that of the pattern observed for st. 40/41 control eye (compare Figure 5B7,B7’ with Figure 5A6,A6’). Müller glial differentiation was restored by st. 45/46 at 4 dps (Figure 5B8, B8’). Together, the data showed that Müller glial differentiation was delayed as compared to the control eye. However, Müller glial differentiation was restored by 4 days – a timepoint that was later than the restoration of patterning observed for other retinal cell types. These findings supported the hypothesis that Müller glial cells are specified later than other retinal cell types in the regrowing eye.

### Changes in Pax6 Expression During Regrowth

**FIGURE 6:**
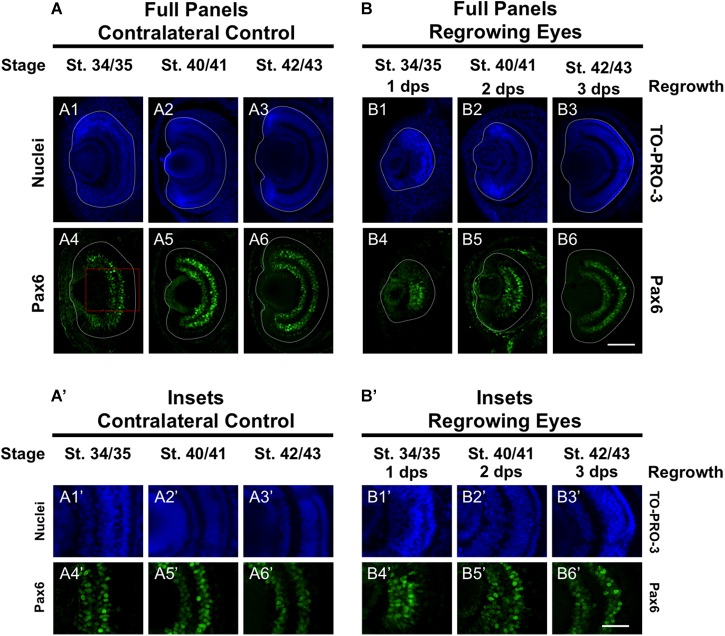
Regrown eyes regain Pax6 patterning by 3 dps. Images shown are immunostained, transverse sections at three developmental timepoints corresponding to 1, 2, and 3 days post surgery (dps). **(A,B)** Pax6 expression in the regrowing eye is less organized at 1 dps but regains patterning similar to contralateral control eyes (unoperated) by 3 dps. White dashed lines delineate each regrowing eye. **(A’,B’)** Images shown in panels **A’** and **B’** correspond to the region shown in the inset box in panel **A4** for the corresponding **A or B** panel at high magnification. Blue color indicates nuclear staining (TO-PRO-3). Green color indicates anti-Pax6 signal. Sample sizes: 1 day, *n* = 5; 2 days, *n* = 7; and 3 days, *n* = 6. **(A,B, A’,B’)** Up = dorsal, down = ventral, lens is on the left. Scale bar: **A,B** = 100 μm and **A’,B’** = 50 μm.

Pax6 is an eye field transcription factor that is expressed in the presumptive eye primordium after gastrulation (st. 12.5) and specifies the eye field (Zuber et al., 2003). Prior to st. 33/34, Pax6 mRNA is expressed throughout the neural retina (Hirsch and Harris, 1997). By st. 33/34 and onward, Pax6 mRNA expression becomes more restricted to the presumptive GCL and INL of the retina so that by st. 42, Pax6 expression is observed only in those two layers (Hirsch and Harris, 1997). We used an anti-Pax6 antibody to assess its expression during regrowth (Rungger-Brändle et al., 2010). Consistent with previous reports, we observed that Pax6 expression in the control eye was mostly restricted to the presumptive GCL and INL and extended out to the periphery at st. 34/35 (Figure 6A4,A4’). By st. 40/41, Pax6 expression was tightly restricted to the GCL and INL (Figure 6A5,A5’) and retained this expression pattern through st. 42/43 (Figure 6A6,A6’). In the 1 dps regrowing eye, the retinal layers were not apparent (as seen by Xen1 expression, Figure 1B7). At this timepoint, Pax6 expression was not localized and remained expanded, with apparent higher expression levels in the central region (Figure 6B4,B4’). This pattern was more reminiscent of Pax6 expression in embryos younger than st. 33 (Hirsch and Harris, 1997). By 2 dps (st. 40/41), Pax6 was largely restricted to the GCL and INL in the regrowing eye although expression near the retinal periphery is weaker than those cells located more centrally (Figure 6B5,B5’). By 3 dps, Pax6 patterning was restored as its expression became restricted to GCL and INL (Figure 5B6,B6’). Together, our data indicated that Pax6 expression was not restricted to the GCL and INL layers 1 dps in the regrowing eye. As regrowth continues, these Pax6-expressing cells changed and became restricted to the GCL and INL of the retina by 3 dps.

### Assessment of the Roles of Pax6 and Apoptosis During Development and Regrowth

A key feature of this developmental eye repair model is that it can facilitate a rapid assessment of development and regenerative mechanisms. Our previous work and current data combined suggest that eye formation and differentiation during regrowth is delayed but largely followed the normal developmental process, resulting in an eye that was indistinguishable to a normal one (Figure 1–6; Kha et al., 2018). This model now provides the opportunity to use the same developmental context to ask whether specific molecular mechanisms are required in development and/or regeneration for the eye. Therefore, we assessed the roles of Pax6 (which is required for eye development) and apoptosis (which is required for eye regrowth) in both eye development and regrowth.

**FIGURE 7:**
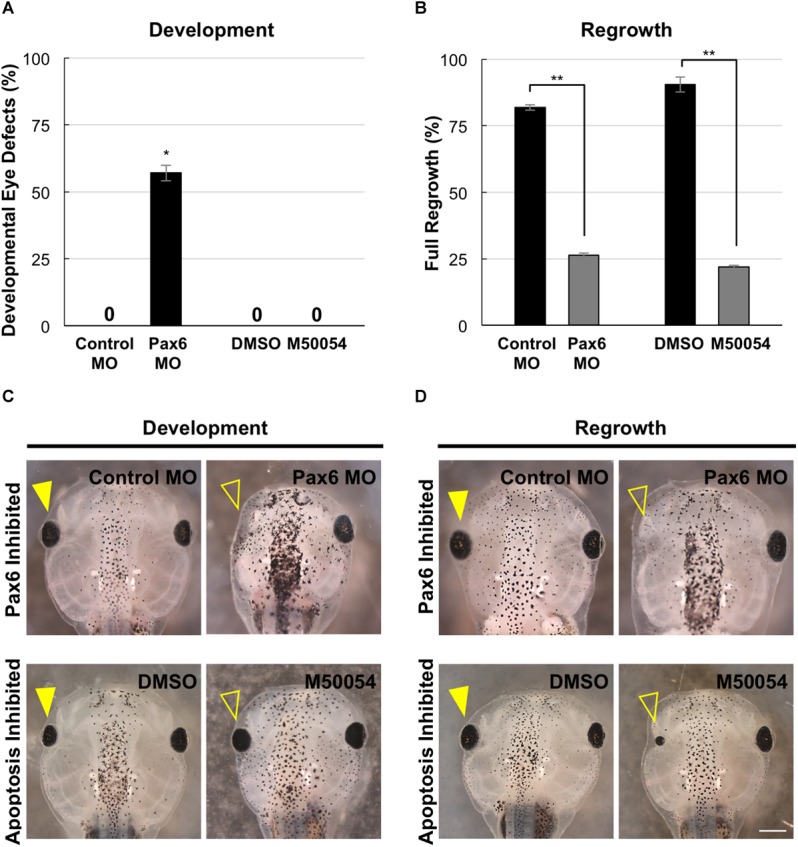
Development and regrowth require Pax6, but only regrowth requires apoptosis. **(A)** Comparison of developmental eye defects percentage from embryos injected at the 4-cell stage (1 blastomere) with either the control or Pax6 morpholino or treated with DMSO control or M50054 at st. 10. A zero denotes no abnormal phenotype in the control by st. 27. ^∗^denotes *p* < 0.05 (*n* > 90). Data are means ± SEM. **(B)** Graphical representation of tadpoles achieving full eye regrowth at 5 dps (st. 46) with morpholino injection or apoptosis inhibitor treatment. ^∗∗^denotes *p* < 0.01 (*n* > 20). Data are means ± SEM. **(C,D)** Comparison of requirements for eye development and regrowth. **(C)** Pax6 morpholino injected tadpoles show reduced eyes when compared to the control by st. 46 in development. Apoptosis inhibitor show no effect on eye development (*n* > 30 per condition). Closed yellow arrowhead indicates eye of control, untreated tadpole. Open yellow arrowhead indicates eye of treated tadpole. **(D)** Pax6 morpholino and apoptosis inhibitor affects eye regrowth (*n* > 30). Closed yellow arrowhead indicates the eye of a control, untreated tadpole. Open yellow arrowhead indicates the eye of an inhibitor treated tadpole. **(C,D)** Up = anterior, down = posterior. Scale bar: **C,D** = 500 μm.

Pax6 is required for proper vertebrate eye development. In *Xenopus tropicalis*, loss-of-function Pax6 mutations reduced eye size and shows additional eye defects (Nakayama et al., 2015). *X. laevis* embryos injected with a Pax6 morpholino showed reduced or absent eyes (Rungger-Brändle et al., 2010). We also examined Pax6 loss-of-function effects on the eye. We injected either a published Pax6 morpholino or a control morpholino into the dorsal blastomere at the 4-cell stage and assessed for eye defects at a tadpole stage (st. 46). Consistent with previous studies, Pax6 morpholino expression resulted in eye defects in the majority of embryos (57.1%, *n* = 91) as compared to embryos expressing a control morpholino (0%, *n* = 30, *p* < 0.05) (Figure 7A). The eye defects included reduced or absent eyes (Figure 7C, compare top panels).

In *X. laevis*, apoptosis can be detected in embryos starting at gastrulation (st. 10.5) and was observed in the anterior region throughout neurulation (Hensey and Gautier, 1998). For apoptosis inhibition during development, we used M50054, a known apoptosis inhibitor that blocks caspase activity and successfully inhibited both *Xenopus* tadpole tail regeneration and eye regrowth (Tsuda et al., 2001, Tseng et al., 2007; Kha et al., 2018). Embryos were treated with 28 μM of M50054 from st. 10 (gastrulation) to st. 27 (tailbud embryo) and scored at st. 46 (tadpole). Embryos treated with either M50054 (*n* = 30) or DMSO (vehicle only, *n* = 30), did not display any morphological eye defects (Figure 7A,C, compare bottom panels). Our previous study also showed that M50054 treatment from st. 27 to st. 34/35 did not induce eye defects (Kha et al., 2018). These data were also consistent with a previous study showing that overexpression of the anti-apoptotic gene, BcL-xL, during embryogenesis did not induce eye defects (Johnston et al., 2005). Thus, apoptosis does not appear to be required for eye development.

To assess the role of Pax6 in eye regrowth, the same Pax6 morpholino injection was carried out using a reduced concentration so as to enable normal overall development. This is to ensure that eye tissue removal surgery can be performed on embryos with normal eyes. 81.8% of embryos expressing the control morpholino in the eye region at st. 27 fully regrew eyes (Figure 7B, RI = 278, *n* = 22; and Figure 7D, compare top panels). In contrast, only 13.7% of embryos expressing the Pax6 morpholino in the eye region at st. 27 showed full eye regrowth whereas 86.3% failed (Figure 7B, RI = 168, *n* = 51, *p* < 0.01 when compared to control; and Figure 7D compare top panels). Thus, Pax6 morpholino successfully blocked eye regrowth. For apoptosis, we confirmed our previous study showing that inhibition of apoptosis using M50054 blocked eye regrowth [Figure 7B, *n* = 41, *p* < 0.01, and Figure 7D; compare bottom panels, and (Kha et al., 2018)]. Our data indicate that Pax6 is required for successful *Xenopus* eye regrowth. Although this is not an unexpected result, this data showed that at least one key eye development gene is used for eye regrowth.

## Discussion

**FIGURE 8:**
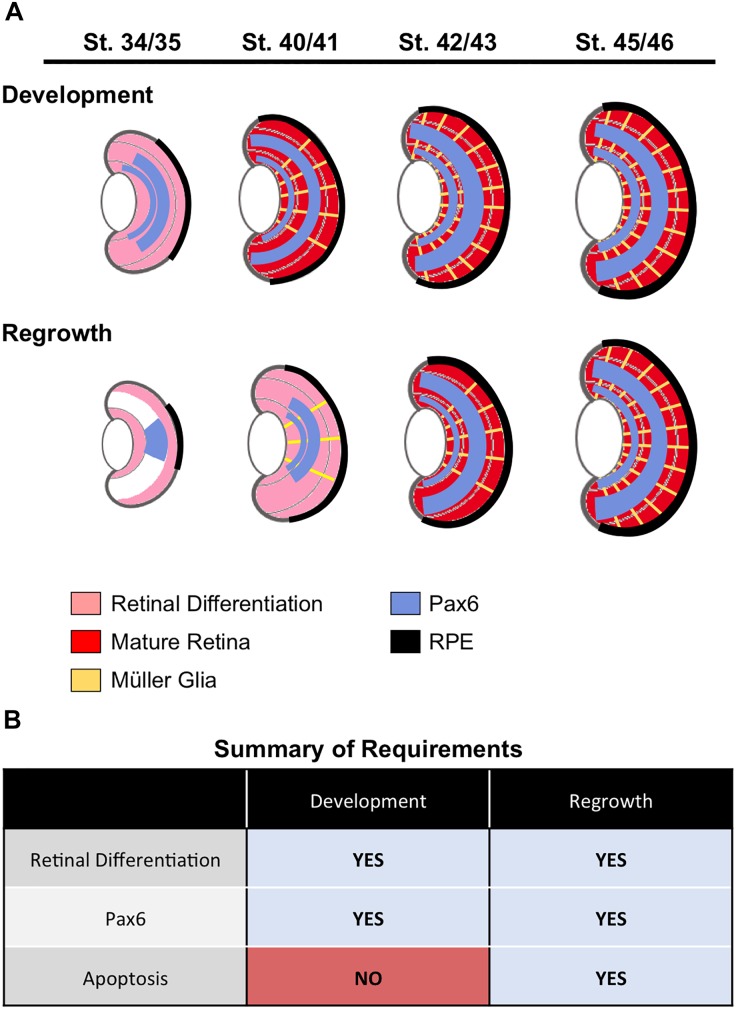
Summary. **(A)** A summary of retinal cell differentiation during regrowth as compared to development. The stages correspond to 1, 2, 3, and 4 days post surgery (dps), respectively. **(B)** Summary of required processes identified in eye development and regrowth.

In this study, we showed that eye formation during regrowth was delayed but generally followed the endogenous retinal differentiation and cellular patterning process to generate a regrown eye that is age and size appropriate (summarized in Figure 8A). Consistent with this data, the formation of the ciliary margin zone (CMZ) was also delayed. The CMZ is located at the periphery of the retina and produces all retinal cell types for eye growth post-embryonically (Hollyfield, 1971). It can be visualized by its distinct spatial cellular organization in eye sections and was formed by st. 34/34 (Supplementary Figures S1A,A’). In the regrowing eye, the formation of the CMZ was delayed until st. 40/41 (2 dps; Supplementary Figures S1B,B’).

A distinct characteristic of retinogenesis is that it contains an intrinsic timer for initiating differentiation. In *X. laevis*, retinogenesis timing remained the same and began by st. 24 even when there was a significant reduction of retinal progenitors cells by chemical inhibition of proliferation during embryogenesis (Harris and Hartenstein, 1991). In our eye regrowth model, a significant reduction of retinal progenitors (average loss is approximately 83%) is achieved by tissue removal surgery at st. 27 (Kha et al., 2018). Here, we examined the temporal regulation of the regrowth process in more detail. Endogenous retinogenesis is initiated at st. 24 and completes by st. 41, a time period of about 2 days (Holt et al., 1988). Our previous work showed there was a significant increase in proliferation at the injury site during the first 24 h of regrowth (Kha et al., 2018). Here, we report that reparative retinogenesis showed a delay and started at 1 dps (st. 34/35) with completion occurring by 3 dps (st. 42/43). Like the endogenous process, reparative retinogenesis needed a time period of about 2 days. These results suggest that while retinogenesis can be re-induced at a developmental stage later than st. 24, the overall time required to complete the differentiation process was maintained as for development. Even though the eye formation time window can be re-started past the endogenous timeframe, there was no shortening of the eye formation period to catch up as quickly as possible.

As the first differentiated retinal cells are generated starting at st. 24, there is a continual decrease in the mitotic index of the retinal progenitor cells (RPCs) until most cells have exited the cell cycle by st. 37/38 (Holt et al., 1988). During this time, the estimated cell doubling time increases from 8.6 to 56 h (Rapaport, 2006). In contrast, there is significantly increased mitotic activity in the first 24 h during eye regrowth that continues until the regrown eye reached the expected age-appropriate size by 3 dps (Kha et al., 2018). The proliferative burst of RPCs in eye regrowth is counter to the endogenous process at the same developmental stages where cells are becoming postmitotic. Moreover, the increase in RPC proliferation, coupled with the delay of retinal differentiation suggests that induction of regrowth temporarily inhibited retinogenesis. There is no specific cell number required for retinogenesis as the initiation of *Xenopus* retinogenesis is not affected by greatly reduced retinal cell divisions during embryogenesis (Harris and Hartenstein, 1991). One possibility is that the sudden loss of RPCs at st. 27 via tissue removal surgery triggers a signal that extends the stem cell multipotency of RPCs in order to restore normal size. (It is also possible that the source cells may be non-retinal in origin.) Although we used Pax6 as a differentiation marker in this study, it is also required for maintaining the multipotent state of RPCs prior to retinogenesis (Marquardt et al., 2001). The absence of restricted Pax6 expression in the regrowing eye at 1 dps (compare Figure 6B4,A4) is reminiscent of its expression at the younger, proliferative, developmental stages (Hirsch and Harris, 1997). It will be highly informative to identify the molecular mechanisms that regulate RPC proliferation during regrowth as this model has the potential to become a useful system to study endogenous RPC expansion.

During eye regrowth (st. 27 to st. 42/43), the embryo is changing rapidly as it proceeds from being a tailbud embryo with unformed organs toward becoming a tadpole with differentiated body structures (Nieuwkoop and Faber, 1994). Once eye regrowth is initiated after tissue loss, it appears to follow the endogenous developmental program and remain unaffected by rapid changes in the surrounding tissues during development. Indeed, the overall retinal birth order that was observed for the cell types examined was consistent with the described order for *Xenopus* retinogenesis. Of note, our study of retinal differentiation during regrowth did not specifically examine each individual retinal cell type that is generated during eye formation. It is possible that there may exist some differences in formation of the regrown eye as compared to eye development that was not detected by the retinal markers used in this study.

Our findings revealed that successful eye development during regrowth induced similar cellular events as for eye development. This model now provides the opportunity to directly examine the role of developmental mechanisms in eye regrowth. We used this model to compare the role of two mechanisms, Pax6 and apoptosis, in development and regrowth (summarized in Figure 8B). Given the role of Pax6 as a “master regulator” of eye formation, it was not surprising that Pax6 was found to be also required for eye regrowth. In contrast, we found that apoptosis appears to be a regrowth-specific mechanism. Thus we have successfully used this model to define an initial similarity and an initial difference between eye development and regrowth. As there is a wealth of knowledge on the role of Pax6 (and other known regulators) during eye development, it will be highly feasible to distinguish any differences in the function of Pax6 and other genes in regrowth. For further comparison to developmental eye regrowth, follow-up studies can then be performed to examine the role of these genes in tadpole and adult retinal regeneration using established *Xenopus* models (Yoshii et al., 2007; Vergara and Del Rio-Tsonis, 2009; Araki, 2014). Potentially, developmental mechanisms that are not required for eye regrowth can also be identified. In summary, this developmental eye regrowth model will serve as a robust platform for systematically examining the common view that regeneration is a recapitulation of development.

## Materials and Methods

### Embryo Culture and Surgery

Embryos were obtained via *in vitro* fertilization and raised in 0.1× Marc’s Modified Ringer (MMR: 1 mM MgSO_4_, 2.0 mM KCl, 2 mM CaCl_2_, 0.1 M NaCl, 5 mM HEPES, pH 7.8) medium (Sive et al., 2000). The eye removal surgery and the regrowth assay were performed as described previously (Kha et al., 2018). Embryos at stage (st.) 27 (Nieuwkoop and Faber, 1994) were anesthetized with MS222 (Sigma) prior to surgery. Surgery was performed using fine surgical forceps (Dumont No. 5). An initial cut is first made in the skin surrounding the protruding eye cup and overlying lens placode. The cut is continued around the raised outline of the eye and the protruding tissues are removed. After surgery, embryos were transferred into 0.1× MMR, allowed to recover, and then cultured at 22°C for 1–5 days.

### Embryo Sectioning and Immunofluorescence Microscopy

For agarose embedding and sectioning, animals were fixed overnight at 4°C in MEMFA (100 mM MOPS (pH 7.4), 2 mM EGTA, 1 mM MgSO_4_, 3.7% (v/v) formaldehyde) (Sive et al., 2000) and processed according to Kha et al. (2018). Embryos and tadpoles were embedded in 4–6% low-melt agarose and sectioned into 60 μm slices using a Leica vt1000s vibratome. Sections were stained with primary antibodies including: Xen1 (pan-neural antibody, clone 3B1, 1:50 dilution, Developmental Studies Hybridoma Bank, **RRID: AB_531871**), anti-Islet1 (retinal ganglion cells and inner nuclear cell layer, clone 40.2D6, 1:200 dilution, Developmental Studies Hybridoma Bank, **RRID: AB_528315**), anti-Glutamine Synthetase (Müller glia, 1:200 dilution, Sigma-Aldrich, **RRID: AB_259853**), anti-Laminin (basal lamina, 1:300 dilution, Sigma-Aldrich, **RRID: AB_477163**), anti-Rhodopsin (rod photoreceptor cells, clone 4D2, 1:200 dilution, EMD Millipore, **RRID: AB_10807045**), anti-Calbindin-D-28 K (cone photoreceptor cells, 1:500 dilution, Millipore Sigma, **RRID: AB_258818**), anti-Pax6 (clone Poly19013, 1:500 dilution, BioLegend, **RRID: AB_291612**), anti-RPE65 (retinal pigment epithelium, 1:500 dilution, ThermoFisher Scientific, **RRID: AB_2181003**). Alexa fluor conjugated secondary antibodies were used at 1:1000 dilution (ThermoFisher Scientific). TO-PRO-3 (Molecular Probes) was used for DNA staining. The contralateral eye was used as the control. For each timepoint, at least 5 embryos were analyzed. In all embryos examined, the observed cellular patterns were consistent for each antibody that was used. Quantification of rod photoreceptor cell numbers was performed using sections stained with an anti-Rhodopsin antibody. The number of rod photoreceptor cells was counted per 60 μm sections (*n* > 5 per timepoint). Rod photoreceptor cells expression pattern was measured in pixels as a drawn line along the outer nuclear layer and compared to the overall circumference of the retinal layer from one end of the ciliary margin zone (CMZ) to the other end of the CMZ (*n* > 5 per timepoint). The ratio of rhodopsin expression in the retinal layer over corresponding the retinal layer circumference measurement was calculated.

### Microscopy

A Nikon A1R confocal laser scanning microscope (UNLV Confocal and Biological Imaging Core) was used to image immunostained tissue sections. Images of whole animals were obtained using a ZEISS SteREO Discovery V20 microscope with an AxioCam MRc camera. ZEN Image Analysis software and/or the open-source FIJI imaging software (Schindelin et al., 2012) were used to analyze and/or process all acquired images.

### Chemical Treatments and Morpholino Injections

For apoptosis inhibition, embryos were treated with 28 μM of M50054 (Millipore, EMD Biosciences, Burlington, MA, United States, CAS number 54135-60-3). For vehicle control, dimethyl sulfoxide (DMSO) was used at the same concentration as for M50054 treatment (0.1%). For the developmental assay, age-matched embryos were raised in 0.1× MMR medium containing the inhibitor starting at st. 10 until st. 27. Eye development was assayed by st. 46. To assay for regrowth, eye surgery was performed on st. 27 tailbud embryos. The embryos were allowed to briefly recover, and then transferred into 0.1× MMR medium containing the inhibitor. After 1 day, embryos were washed with two changes of 0.1× MMR. Eye regrowth was assayed between 1 and 5 days post-surgery.

For morpholino injections, the following morpholinos (MO) were purchased from Gene Tools LLC (Philomath, Oregon): Pax6MO: 5′-GCTGTGACTGTTCTGCATGTCGAG-3′ (Li et al., 1997; Rungger-Brändle et al., 2010); and the non-specific standard control oligomer: 5′-CCTCTTACCTCAGTTACAATTTATA-3′. Each morpholino was modified with 3′ fluorescein. Morpholinos were resuspended in sterile water to a concentration of 1 mM. For both developmental and regrowth studies, morpholinos were injected separately into a dorsal blastomere of a 4-cell embryo using a microinjector (Harvard Apparatus, Holliston, MA)– targeting only one side of the embryo. Embryos with fluorescent signal in the eye region were selected for further analysis. A previously published concentration of 30 ng/embryo (Rungger-Brändle et al., 2010) was used for verification of published phenotypes. The titrated dosages for morpholino injections were: 27 ng/embryo (developmental assay) and 15 ng/embryo (eye regrowth assay). Lethality was observed in st. 27 tailbud embryos that were injected with 35.7 ng of Pax6 morpholino at the 4-cell stage.

### Assessment of Eye Regrowth

The regrowth of the operated eyes as compared to unoperated contralateral eyes was assessed using the Regrowth Index (RI) as previously described (Kha et al., 2018). The quality of eye regrowth was scored based on 4 phenotype categories: full, good, weak, and none. Full, RI = 300; Partial, RI = 200; Weak, RI = 100; None, RI = 0. The RI ranges from 0 to 300, where 0 indicates no eye regrowth of all embryos in a given condition, 100 if all embryos achieve weak regrowth, 200 if all embryos achieve good regrowth, and 300 indicates that all embryos achieve full regrowth. Raw data from scoring was used to compare eye regrowth experiments. The unoperated contralateral eyes of embryos showed no difference from unoperated control eye of age-matched sibling embryos.

### Statistical Analysis

To compare eye regrowth, raw data from scoring was used. Comparison of two treatments was analyzed with Mann-Whitney *U* test for ordinal data with tied ranks, using normal approximation for large sample sizes. Multiple treatments were compared using a Kruskal-Wallis test, with Dunn’s Q corrected for tied ranks. All other experiments were analyzed using a Student’s *t*-test.

## Ethics Statement

This study was carried out in accordance with the recommendations of the University of Nevada, Las Vegas Institutional Animal Care and Use Committee (IACUC). The protocol was approved by the UNLV IACUC.

## Author Contributions

KT contributed the conception and design of the study. CK and DG performed the experiments. CK, DG, and KT analyzed the data, wrote, revised, and approved the manuscript.

## Conflict of Interest Statement

The authors declare that the research was conducted in the absence of any commercial or financial relationships that could be construed as a potential conflict of interest.

## References

[B1] Álvarez-Hernán G. Bejarano-Escobar R. Morona R. González A. Martín-Partido G. Francisco-Morcillo J. (2013). Islet-1 immunoreactivity in the developing retina of *Xenopus laevis*. *Sci. World J.* 2013:740420. 10.1155/2013/740420 24348185 PMC3844241

[B2] Araki M. (2007). Regeneration of the amphibian retina: role of tissue interaction and related signaling molecules on RPE transdifferentiation. *Dev. Growth Differ.* 49 109–120. 10.1111/j.1440-169x.2007.00911.x 17335432

[B3] Araki M. (2014). “A model for retinal regeneration in *Xenopus*,” in *Xenopus Development*, eds Kloc M. Kubiak J. Z. (New York, NY: Oxford University Press), 346–367. 10.1002/9781118492833.ch18

[B4] Beck C. W. Izpisúa Belmonte J. C. Christen B. (2009). Beyond early development: *Xenopus* as an emerging model for the study of regenerative mechanisms. *Dev. Dyn.* 238 1226–1248. 10.1002/dvdy.21890 19280606

[B5] Dorsky R. I. Chang W. S. Rapaport D. H. Harris W. A. (1997). Regulation of neuronal diversity in the *Xenopus retina* by Delta signalling. *Nature* 385 67–70. 10.1038/385067a0 8985247

[B6] Halasi G. Søviknes A. M. Sigurjonsson O. Glover J. C. (2012). Proliferation and recapitulation of developmental patterning associated with regulative regeneration of the spinal cord neural tube. *Dev. Biol.* 365 118–132. 10.1016/j.ydbio.2012.02.012 22370002

[B7] Harris W. A. Hartenstein V. (1991). Neuronal determination without cell division in *Xenopus* embryos. *Neuron* 6 499–515. 10.1016/0896-6273(91)90053-3 1901716

[B8] Henry J. J. Thomas A. G. Hamilton P. W. Moore L. Perry K. J. (2013). “Cell signaling pathways in vertebrate lens regeneration,” in *New Perspectives in Regeneration*, eds Heber-Katz E. Stocum D. L. (Berlin: Springer), 75–98. 10.1007/82_2012_289 PMC4304700 23224710

[B9] Henry J. J. Wever J. M. Natalia Vergara M. Fukui L. (2008). “*Xenopus*, an ideal vertebrate system for studies of eye development and regeneration,” in *Animal Models in Eye Research*, ed. Tsonis P. A. (San Diego, CA: Academic Press), 57–92. 10.1016/b978-0-12-374169-1.00006-0

[B10] Hensey C. Gautier J. (1998). Programmed cell death during *Xenopus* development: a spatio-temporal analysis. *Dev. Biol.* 203 36–48. 10.1006/dbio.1998.9028 9806771

[B11] Hirsch N. Harris W. A. (1997). *Xenopus* Pax-6 and retinal development. *J. Neurobiol.* 32 45–61. 10.1002/(sici)1097-4695(199701)32:1<45::aid-neu5>3.0.co;2-e 8989662

[B12] Hollyfield J. G. (1971). Differential growth of the neural retina in *Xenopus laevis* larvae. *Dev. Biol.* 24 264–286. 10.1016/0012-1606(71)90098-4 5553368

[B13] Holt C. E. Bertsch T. W. Ellis H. M. Harris W. A. (1988). Cellular determination in the *Xenopus retina* is independent of lineage and birth date. *Neuron* 1 15–26. 10.1016/0896-6273(88)90205-x 3272153

[B14] Johnston J. Chan R. Calderon-Segura M. McFarlane S. Browder L. W. (2005). The roles of Bcl-xL in modulating apoptosis during development of *Xenopus laevis*. *BMC Dev. Biol.* 5:20. 10.1186/1471-213X-5-20 16185362 PMC1262703

[B15] Kha C. X. Son P. H. Lauper J. Tseng K. A.-S. (2018). A model for investigating developmental eye repair in *Xenopus laevis*. *Exp. Eye Res.* 169 38–47. 10.1016/j.exer.2018.01.007 29357285

[B16] Kha C. X. Tseng K. A.-S. (2018). Developmental dependence for functional eye regrowth in *Xenopus laevis*. *Neural Regen. Res.* 13 1735–1737. 30136686 10.4103/1673-5374.238611 PMC6128050

[B17] Li H. Tierney C. Wen L. Wu J. Y. Rao Y. (1997). A single morphogenetic field gives rise to two retina primordia under the influence of the prechordal plate. *Development* 124 603–615. 9043075 10.1242/dev.124.3.603 PMC2041934

[B18] Lin G. Slack J. M. W. (2008). Requirement for Wnt and FGF signaling in *Xenopus tadpole* tail regeneration. *Dev. Biol.* 316 323–335. 10.1016/j.ydbio.2008.01.032 18329638

[B19] Malloch E. L. Perry K. J. Fukui L. Johnson V. R. Wever J. Beck C. W. (2009). Gene expression profiles of lens regeneration and development in *Xenopus laevis*. *Dev. Dyn.* 238 2340–2356. 10.1002/dvdy.21998 19681139 PMC2773617

[B20] Marquardt T. Ashery-Padan R. Andrejewski N. Scardigli R. Guillemot F. Gruss P. (2001). Pax6 is required for the multipotent state of retinal progenitor cells. *Cell* 105 43–55. 10.1016/s0092-8674(01)00295-1 11301001

[B21] Martinez-De Luna R. I. Kelly L. E. El-Hodiri H. M. (2011). The Retinal Homeobox (Rx) gene is necessary for retinal regeneration. *Dev. Biol.* 353 10–18. 10.1016/j.ydbio.2011.02.008 21334323 PMC3093306

[B22] Meyers J. R. Hu L. Moses A. Kaboli K. Papandrea A. Raymond P. A. (2012). β-catenin/Wnt signaling controls progenitor fate in the developing and regenerating zebrafish retina. *Neural Dev.* 7:30. 10.1186/1749-8104-7-30 22920725 PMC3549768

[B23] Nakayama T. Fisher M. Nakajima K. Odeleye A. O. Zimmerman K. B. Fish M. B. (2015). Xenopus pax6 mutants affect eye development and other organ systems, and have phenotypic similarities to human aniridia patients. *Dev. Biol.* 408 328–344. 10.1016/j.ydbio.2015.02.012 25724657 PMC4549229

[B24] Nieuwkoop P. D. Faber J. (1994). *Normal Table of Xenopus laevis (Daudin) : A Systematical and Chronological Survey of the Development from the Fertilized Egg Till the End of Metamorphosis*. New York, NY: Garland Publishing.

[B25] Perron M. Harris W. A. (1999). “Cellular determination in amphibian retina,” in *Cell Lineage and Fate Determination*, 1st Edn, ed. Moody S. A. (New York: Academic Press), 353–368. 10.1016/b978-012505255-9/50024-9

[B26] Rapaport D. H. (2006). “Retinal neurogenesis,” in *Retinal Development*, eds Sernagor E. Eglen S. Harris B. Wong R. (Cambridge, MA: Cambridge University Press), 30–58. 10.1017/cbo9780511541629.005

[B27] Ruiz i Altaba A. (1992). Planar and vertical signals in the induction and patterning of the *Xenopus* nervous-system. *Development* 116 67–80. 1483396 10.1242/dev.116.1.67

[B28] Rungger-Brändle E. Ripperger J. A. Steiner K. Conti A. Stieger A. Soltanieh S. (2010). Retinal patterning by Pax6-dependent cell adhesion molecules. *Dev. Neurobio* 70 764–780. 10.1002/dneu.20816 20556827

[B29] Sater A. K. Moody S. A. (2017). Using *Xenopus* to understand human disease and developmental disorders. *Genesis* 55:e22997. 10.1002/dvg.22997 28095616

[B30] Schaefer J. J. Oliver G. Henry J. J. (1999). Conservation of gene expression during embryonic lens formation and cornea-lens transdifferentiation in *Xenopus laevis*. *Dev. Dyn.* 215 308–318. 10.1002/(sici)1097-0177(199908)215:4<308::aid-aja3>3.3.co;2-9 10417820

[B31] Schindelin J. Arganda-Carreras I. Frise E. Kaynig V. Longair M. Pietzsch T. (2012). Fiji: an open-source platform for biological-image analysis. *Nat. Methods* 9 676–682. 10.1038/nmeth.2019 22743772 PMC3855844

[B32] Sive H. L. Grainger R. M. Harland R. M. (2000). *Early Development of Xenopus laevis: A Laboratory Manual*. Cold Spring Harbor, NY: Cold Spring Harbor Laboratory Press.

[B33] Tseng A.-S. (2017). Seeing the future: using *Xenopus* to understand eye regeneration. *Genesis* 55:e23003. 10.1002/dvg.23003 28095643

[B34] Tseng A.-S. Adams D. S. Qiu D. Koustubhan P. Levin M. (2007). Apoptosis is required during early stages of tail regeneration in *Xenopus laevis*. *Dev. Biol.* 301 62–69. 10.1016/j.ydbio.2006.10.048 17150209 PMC3136124

[B35] Tsuda T. Ohmori Y. Muramatsu H. Hosaka Y. Takiguchi K. Saitoh F. (2001). Inhibitory effect of M50054, a novel inhibitor of apoptosis, on anti-Fas-antibody-induced hepatitis and chemotherapy-induced alopecia. *Eur. J. Pharmacol.* 433 37–45. 10.1016/s0014-2999(01)01489-3 11755132

[B36] Vergara M. N. Del Rio-Tsonis K. (2009). Retinal regeneration in the *Xenopus laevis* tadpole: a new model system. *Mol. Vis.* 15 1000–1013. 19461929 PMC2684558

[B37] Viczian A. S. Zuber M. E. (2015). “Retinal development,” in *Principles of Developmental Genetics*, ed. Moody S. A. (London: Elsevier), 297–313.

[B38] Wang J. Conboy I. (2010). Embryonic vs. adult myogenesis: challenging the “regeneration recapitulates development” paradigm. *J. Mol. Cell Biol.* 2 1–4. 10.1093/jmcb/mjp027 19797316

[B39] Wong L. L. Rapaport D. H. (2009). Defining retinal progenitor cell competence in *Xenopus laevis* by clonal analysis. *Development* 136 1707–1715. 10.1242/dev.027607 19395642 PMC2673759

[B40] Yoshii C. Ueda Y. Okamoto M. Araki M. (2007). Neural retinal regeneration in the anuran amphibian *Xenopus laevis* post-metamorphosis: transdifferentiation of retinal pigmented epithelium regenerates the neural retina. *Dev. Biol.* 303 45–56. 10.1016/j.ydbio.2006.11.024 17184765

[B41] Zuber M. E. Gestri G. Viczian A. S. Barsacchi G. Harris W. A. (2003). Specification of the vertebrate eye by a network of eye field transcription factors. *Development* 130 5155–5167. 10.1242/dev.00723 12944429

