# Supplementary figures and images for: Using the Xenopus Developmental Eye Regrowth System to Distinguish the Role of Developmental Versus Regenerative Mechanisms
Source: Front Physiol. 2019 May 8;10:502. doi: 10.3389/fphys.2019.00502 (PMC6518849; doi:10.3389/fphys.2019.00502)

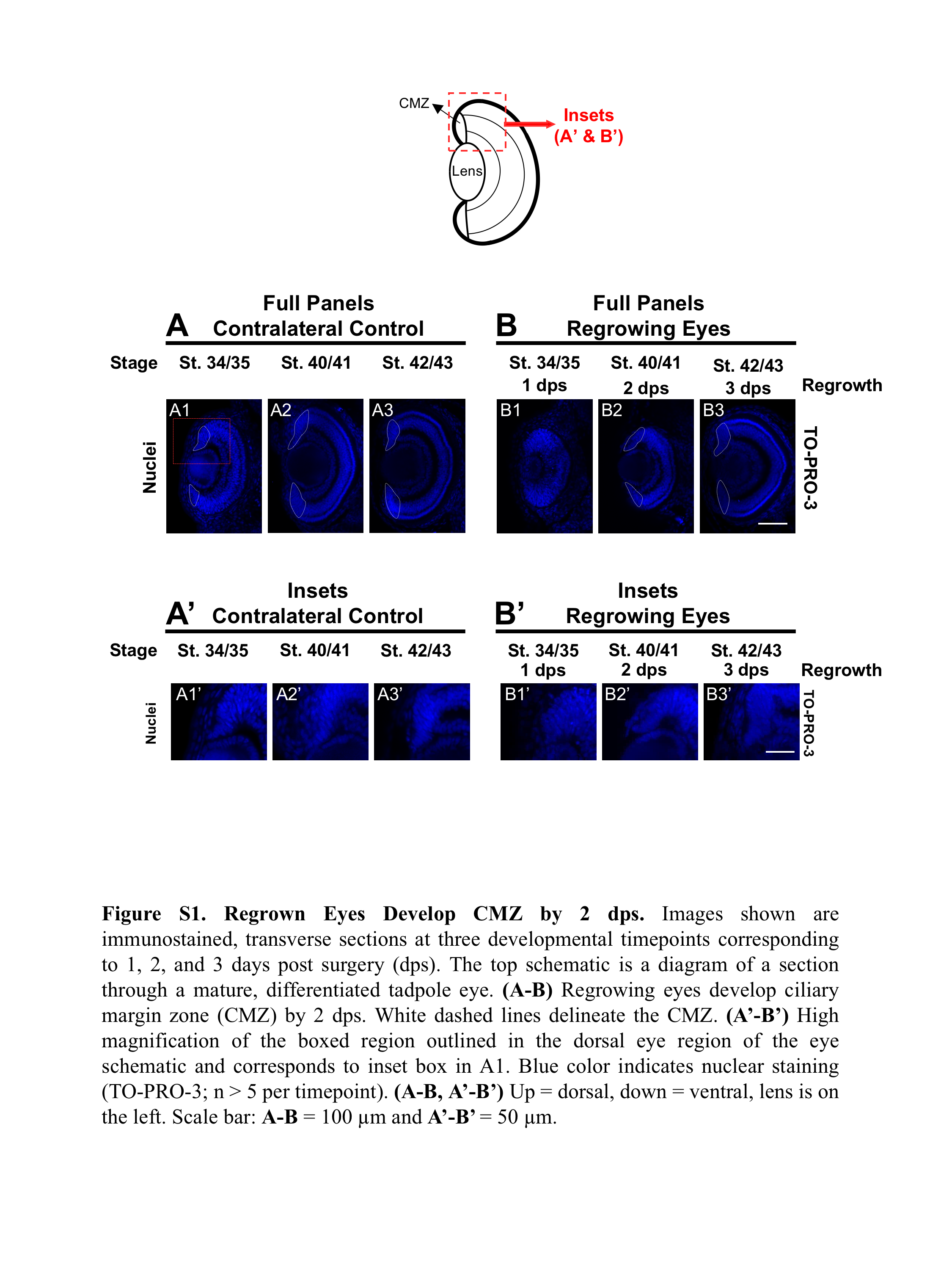

Supplement: Supplementary file 1 [file Image_1.TIFF]
